# Clinical Picture Of Arrhythmogenic Right Ventricular Dysplasia / Cardiomyopathy Patients From Indian Origin

**Published:** 2009-01-07

**Authors:** DVN Maithili, Pranathi Rao Pamuru, Khalid Mohiuddin, Sushant Remersu, Narasimhan Calambur, Sai Satish Oruganti, Pratibha Nallari

**Affiliations:** 1 Department of Genetics,Osmania University, Hyderabad, India; 2 Kakatiya Medical College, Warangal, Hyderabad, India; 3 Cardiology Unit, Care Hospital, The Institute of Medical Sciences, Nampally,Hyderabad, India; 4 Cardiology Unit, Nizams Institute of Medical Sciences, Punjagutta, Hyderabad

**Keywords:** Cardiomyopathies, ARVD/C, Clinical heterogeneity

## Abstract

**Objective:**

Among the inherited cardiomyopathies, Arrhythmogenic right ventricular dysplasia/cardiomyopathy is unique with a peculiar pathology of fibro-fatty replacement. Studies have been carried out all over the world and several groups have reported clinical heterogeneity in manifestation of ARVD/C related symptoms. Present study is an attempt to identify the clinical profile of ARVD/C patients from Asian Indian origin.

**Methods:**

31 patients in the span of three years were diagnosed with ARVD/C. Diagnosis was based on proposed task force criteria.

**Results:**

The mean age at diagnosis was 32.9 ± 16.4 years with slight tilt in male to female ratio (1.46). About 80% cases had palpitations, syncope in 45.16% and dyspnea in 22.5%, whereas 16% of patients were asymptomatic. About 50% of patients revealed a family history of confirmed ARVD/C or sudden death of a family member without any known cause. ECG showed T-wave inversion in about 60% cases, prolongation of QRS was observed in 20% cases. RV dilatation was observed in 80% of patients and 66.7% showed systolic dysfunction. RV free wall motion abnormalities were found in 33% patients. Most of the early onset cases with less than 30 years of age showed family history indicative of ARVD/C. Familial study in three patients indicated early onset of condition in younger generations in two families.

**Conclusions:**

ARVD/C in India shows relatively early age at onset when compared with other Asian populations with more than half of patients showing the disease below the age of 30 years. History in most of the early onset cases revealed family history indicating strong genetic influence.

## Introduction

While Arrhythmogenic right ventricular dysplasia/cardiomyopathy (ARVD/C) is one of the rare forms of cardiomyopathies, it is the second most common cause of sudden death of young adults world-wide [1]. Presenting symptoms include ventricular arrhythmias, manifested as palpitations, sustained ventricular tachycardia, pre-syncope (light-headedness), blackout or syncope, heart failure and even sudden death in some cases. But the non-specific signs may include abdominal pain, decreased exercise tolerance, dizziness, dyspnea (with exertion), fatigue, mental confusion and peripheral edema. The pathological hallmark of this condition is the replacement of right ventricular myocardium with fibro-fatty tissue.

The condition has been reported to have genetic etiology, with about seven genes already identified to carry mutations. Along with the genetic heterogeneity, ARVD/C shows clinical heterogeneity, where a patient may present symptoms of ventricular premature complexes, cardiomegaly or ventricular tachycardia of right ventricular origin in childhood or as an adult [2]. Studies carried out so far by various groups across the world have shown significant variability in expression of ARVD/C phenotype in different populations [3-5]. The present report is an attempt to describe the clinical spectrum of this condition in Indian population.

## Methods

Since the incidence of ARVC is unknown in Indian population, no sample size was predetermined at the beginning of the study. All the cases obtained in the span of three years (from 2004 to 2007), viz., 31 ARVD/C cases  reported at Care Hospitals and National Institute of Medical Sciences (NIMS) Hyderabad, India were included in the study. Among these, 4 have ICD implanted.  Parameters such as age, age at onset, presenting symptoms, electrocardiogaphic and echocardiographic features and the compliance of these patients with proposed task force criteria [6] for confirmed diagnosis of ARVD/C  were analysed. According to the standardized criteria a patient is confirmed with ARVC, if he/she satisfies two major criteria; one major and two minor criteria or four minor criteria.

Informed consent of all the patients participating in the study was obtained along with Institutional Ethics Committee (IEC) approval. An IEC approved data collection form was used to obtain all the parameters required for diagnosis [6]. ECG and Echocardiography and other diagnostic tests were performed following the protocols described elsewhere [8].

Based on the questionnaire provided in the data collection form and the results of the diagnostic tests, patients were classified into four clinico-pathologic stages [7]. The first among the four phases is '***Concealed Phase or Silent Phase***'. Though this phase seems to be early in the process of disease progression, the risk for sudden death persists, especially during extreme exertion. This phase is followed by ***overt arrhythmic phase*** then by ***global right ventricular dysfunctional phase***. The progression of fibro-fatty replacement at this stage occurs in a diffused manner resulting in isolated right heart failure. The final stage conventionally is believed to involve the left ventricle causing ***bi-ventricular pump failure***.

Since patients were followed up for various durations, from 18 to 72 months, Kaplan Meir survival curves were generated using GraphPad Prism 5.

## Results

### Age, Age at onset, Gender

In the span of three years (2004-2007), 31 ARVD/C cases of Asian-Indian origin were diagnosed with ARVD/C. The age of diagnosis of these patients was extremely variable with the youngest patient being four years of age and the oldest being 64 years old and the mean age of this cohort of patients was 32.9 ± 16.4. The mean time between onset of ARVD/C related symptoms and admission to hospital was about 3 years. Among 31 cases, 19 (61.3%) were males with a slight imbalance in male to female ratio (1.46).

### Symptoms

Among the presenting symptoms, 25 (80.6%) of them experienced palpitations, 7 patients had dyspnea (22.5%), 14 patients had syncope (45.16%), presyncope was present in 4 patients (12.9%), chest pain was there in 7 (22.5%), swelling of limbs in 6 patients (19.3%), and 16.2%, i.e., 5 patients were asymptomatic. These 5 asymptomatic patients were detected with ARVD/C after the sudden death of a relative or after a confirmed ARVD/C case in the family. Though most of the patients experienced the above-described symptoms even during rest, one of the patients who was a 56 year old female experienced dyspnea and presyncope particularly on physical strain like climbing steps. Another 37 year old male consulted hospital after repeatedly experiencing syncope during physical strain. Only one patient in our cohort was involved in intense athletic activity. This patient was 20 year old when there was sudden onset of palpitations and dyspnea during participation in athletics.

### Electrocardiographic studies

**Figure 1:**
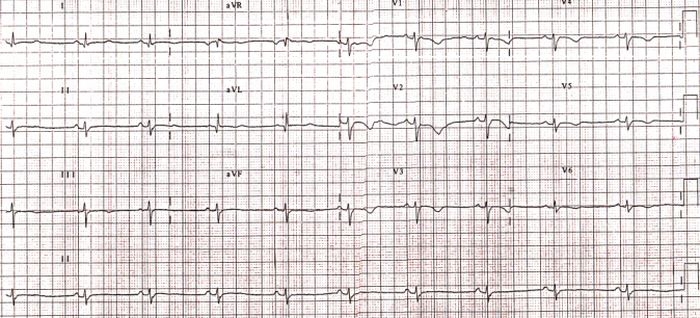
ECG of a patient diagnosed according standardized EFC/ISFC criteria.

12-lead ECG showed sinus rhythm in 74.1% (23) of patients. Most of the patients showed normal (~75 beats per minute) heart rate, 19.3% (6) showed ~60 beats per minute and one patient with ICD showed 50 beats per minute. T-Wave inversions were observed in 61.2% (19) of patients and prolongation of QRS complex, representing prolonged depolarization of heart was observed in 20% (6) of cases. Bundle branch block morphology was observed in 60% (19) of patients, with 22.2% (7) of them showing left bundle branch block (LBBB). Remaining patients showed right bundle branch block morphology (RBBB) reflecting delayed right ventricular activation. ECG was highly sensitive test in the present cohort of patients as 100% cases showed ECG abnormalities indicative of ARVD/C. ECG of a patient (ID-6) is given in Figure 1.

### Echocardiographic changes

**Figure 2:**
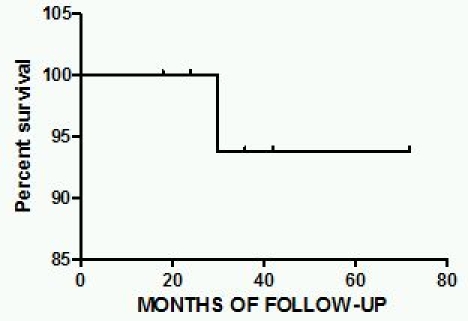

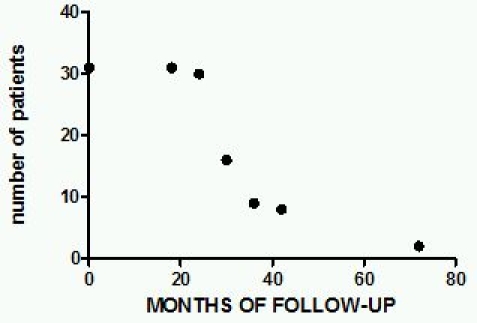
Kaplan Meir survival curve for the cohort of patients included in the present study. The second curve gives the individuals at risk at different points of time. These curves were constructed using GraphPad Prism 5.

Right ventricle was found to be dilated to various degrees (from mild to moderate to severe) in 80% (25) of the patients and systolic dysfunction was observed in 66.7% (21) patients most of them moderately dysfunctional. Tricuspid regurgitation was found to be mild to moderate in 53.4%(17) of patients with out pouchings in right ventricle (RV) in 26.7% (8) and RV free wall motion abnormalities in 33.4% (10) patients. Right atrium was enlarged in 40% (12) of patients but remarkably only one of the patients showed left ventricular involvement. The symptoms and clinical features of the patients has been given in Table 1. All the patients were followed up from 18 to 72 months and during this time the incidence of sudden death was that of 1 patient among 31(0.0322) patients and Kaplan Meir survival curves are shown in Figure 2. 

### Comparison of ARVD/C in early onset and late onset cases

All the 31 patients were divided into two groups, Group A with patients ≤ 30 years and Group B with patients >30 years (Table 2). Number of patients in Group A and Group B were similar (16 and 15 respectively) and distribution of these patients with respect to gender was also similar with 10 males in Group A and 9 males in Group B. All the five asymptomatic patients included in this study were below 30 years of age and therefore belonged to Group A. One of the patients who died during the course of this study was a 10-year-old female, with biventricular involvement and with the family history of ARVD/C (Ped:2), belonging to Group A. Overall, positive family history with either a confirmed case of ARVD/C or with a sudden death of unknown cause was observed in 62.5% (10 patients) in group A and 33.3% (5 patients) in group B. However the difference between the two groups did not show statistical significance (P=0.2666), so a larger sample size is required to corroborate the findings.

### Familial studies

After confirming the diagnosis of ARVD/C, only six of the patients gave consent for screening the family members with most of them refusing to involve other family members probably due to social problems. Among the six patients, three of the families showed positive family history of ARVD/C, where the relatives showed abnormalities in clinical tests but were asymptomatic.

**Figure 3:**
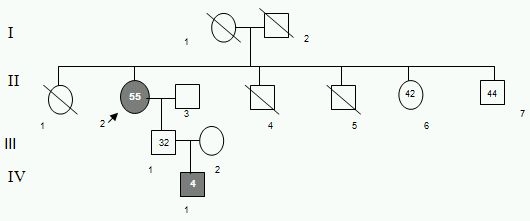
Pedigree 1

First of these patients with family history was a 55 year old female with symptoms of palpitations, syncope, chest pain and swelling of right limbs. The heart showed 50 beats per minute with sinus rhythm and QRS duration of 100 ms and left axis deviation. T-wave inversions were observed in leads V2-V3. This patient was implanted with AICD (automatic implantable defibrillator). Family history revealed sudden death of 2 of her siblings with unknown cause. This patient satisfied 1 major and 2 minor criteria for the confirmation of diagnosis. Screening the available family members, II-3, III-1, III-2 and IV-1 (Figure 3. Ped:1) revealed normal echocardiography and ECG in all of them except IV-1 who was aged 4 years, showed the symptoms of palpitations and on signal averaged ECG (SAECG) revealed premature ventricular complexes (PVCs), revealing the familial nature of the condition in this case.

**Figure 4:**
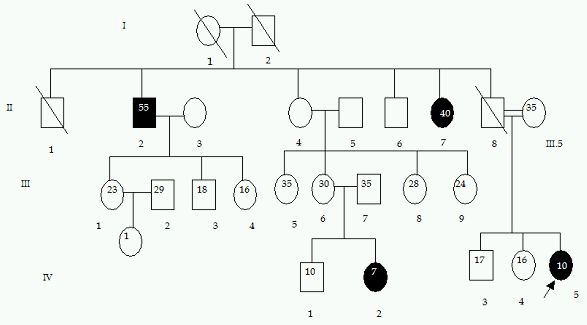
Pedigree 2

Second patient was a 10-year-old girl who had complained of repeated episodes of syncope. After the diagnosis of ARVD/C, the condition was considered to be extremely progressive with biventricular involvement and heart transplantation was being considered within one year of diagnosis as the next treatment option. But she expired during this period even when she was on medication. Clinical examination of the family members revealed three of the relatives, II-2, II-7 and IV-2 satisfying 1- major criteria of confirmed case of ARVD/C in the family and 2 minor criteria of T-wave inversions in right precordial leads and  sudden death of a relative (father of proband (II-8)) at about 30 years of age without any known cause (Figure 4. Ped:2).

**Figure 5:**
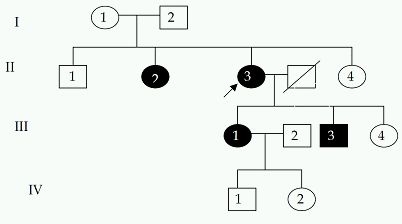
Pedigree 3

Third patient was a 53-year-old female showing the symptoms of dyspnea on exertion (Grade III) and palpitations (Grade II) with the diagnosis being done 2 years ago. Magnetic resonance imaging (MRI) revealed abnormalities of right ventricular morphology and 24 hour Holter showed VT (ventricular tachycardia) from right ventricular outflow tract. Three other members of this family, II-2, III-1 and III-3 were found to have the characteristics of ARVC on clinical examination (Figure 5. Ped:3).

### Classification of patients into clinico-pathologic phases

Among 31 patients included in our study, though 5 patients were asymptomatic, their ECG and 2D-Echocardiography showed gross to mild structural and functional variations. Therefore they could not be classified into the first phase of ***Concealed/Silent phase***, as this phase is characterized by subtle or no structural changes.

23  patients (74%) in the given cohort showed ECG abnormalities such as T-wave inversions characteristic of right ventricular pathology and right ventricle showed echocardiographic variations such as  mild segmental dilatation, localized aneurysms, mild global right ventricular dilatation and also regional right ventricular hypokinesia, all of them having resemblance with  typical ARVD/C hearts.

Seven patients (22.5%) showed features more progressive than the majority of patients leading to right heart failure or severe global right ventricular dysfunction. These patients can be tentatively classified into the third phase of ***Global right ventricular dysfunctional*** phase.

One of the 10 year old patients (Ped: 2) had biventricular involvement with severe dysfunction. This patient can be classified into the final stage.

Overall, all the 31 patients satisfied task force criteria with minimum of 1 major and 2 minor or 2 major or 4 minor criteria.

## Discussion

Previous  reports on clinical manifestation of this condition reveal that ARVD/C shows wide clinical heterogeneity among patients from various geographic areas [2,9,10]. This study is an attempt to describe the clinical manifestations of ARVD/C in Asian-Indian population.

Mean age of onset in the present cohort is 32.9 ± 16.4 years, which  is about a decade earlier than other Asian cohort of patients (Chinese,42 ± 14.8 years and Korean, 41.2 ± 14.8 years) [5,10]. This could be due to the fact that about 50% (16 out of 31) of patients were below 30 years of age and four of these individuals were less that 10 years of age (2 patients 4 years, 1 patient 7 years and 1 patient 10 years of age). This early onset of ARVC could be due to genetic differences between these populations.

Some early studies have reported male preponderance in ARVD/C [11] to the extent of 3:1  and most of the reports on this condition show atleast slight male preponderance. This study shows male to female ratio to be 1.46, slightly infavour of males, which could be due to environmental factors that increase oxidative stress such as tobacco smoking and alcohol, which is predominantly observed in males among Indians. Alternately, this could be due to the  genetic modifying factors that  may predispose males to the condition.

Previous reports on ARVD/C have shown that this condition is the  second most common cause of sudden death in athletes [12]. In the present report, only one patient was a professional athlete at the local district level and only 2 patients experienced symptoms on physical strain. Therefore in this cohort of population ARVD/C is not triggered only during intense physical strain but also is observed in normal population without any athletic activity.

Though ARVD/C is predominantly a right ventricular condition, recent studies implicated increased left ventricular involvement, not only as a pathogenic progression to left ventricle, but also in patients in initial stages of ARVD/C [13,14]. In our study, none of the patients showed predominant left ventricular pathology except in one case where left ventricle was involved after gross right ventricular stages. Other reason for reduced number of patients with left ventricular involvement in present study could be due to the bias in diagnosis, as the patients were detected based only on standardized task force criteria which presently do not include left ventricular pathology.

The comparison of ARVD/C among early onset (below 30 years of age) and late onset (above 30 years of age) cases showed that there was an increased number of asymptomatic cases in early onset group. These patients could not be classified under early stages of ARVD/C because they showed typical pathology of right ventricle such as localized aneurysms, segmental dilation and mild global right ventricular dilatations. This indicates that in these early onset cases, the pathology of right ventricle progresses silently, with out manifestation of any signs of pathogenic condition, that may finally lead to sudden death. Triggering of symptoms may require age-related environmental factors. Further, genetic lesions causing mutations could be playing major role in early onset cases as more than 60% of early onset group indicated familial nature of ARVD/C.

It has been reported that about 50% of ARVD/C cases are familial in nature [11]. In the present study, among 31 patients, 15 patients revealed familial history of sudden death of unknown cause or a confirmed case of ARVD/C. We could not carry out clinical diagnostic tests in all the family members of 31 patients as most of the patients were not willing to participate in the study.

Among the families studied, the age of onset of symptoms was found to earlier in the younger generations compared to the older generations. Also, one of the family was highly asymptomatic in most of the affected individuals. These peculiar features of disease inheritance viz., genetic anticipation and asymptomatic nature of ARVD/C observed in these families could be due to different genetic etiologies, as genetic heterogeneity in ARVD/C is well documented [15].

Though it is not necessary that all the ARVD/C patients would show defined stages in the disease expression, nevertheless, Thiene et al [7] empirically defined the course of ARVD/C into four clinicopathologic phases. Most of the patients (23/31) were under the category of overt arrhythmic phase in which the features of the right ventricle are similar to typical ARVD/C hearts. This also may represent sample bias as most of the patients at this stage are more detectable, than in silent phase or in biventricular failure phase. In biventricular failure phase, it is more likely that the condition is misdiagnosed as dilated cardiomyopathy.

## Conclusion

Our study suggests that ARVC is responsible for cardiac pathology in young Indians. We recommend that individuals with a diagnosis of ARVD have family screening to assess for the presence ARVC in asymptomatic family members. If family screening is routinely performed the incidence of premature SCD will be reduced.

## Limitations

Due to the difficulties associated with the diagnosis of ARVC, there is a chance that many cases obtained during this time could have been misdiagnosed as some other condition and could be responsible for lower sample size.

## Figures and Tables

**Table 1:**
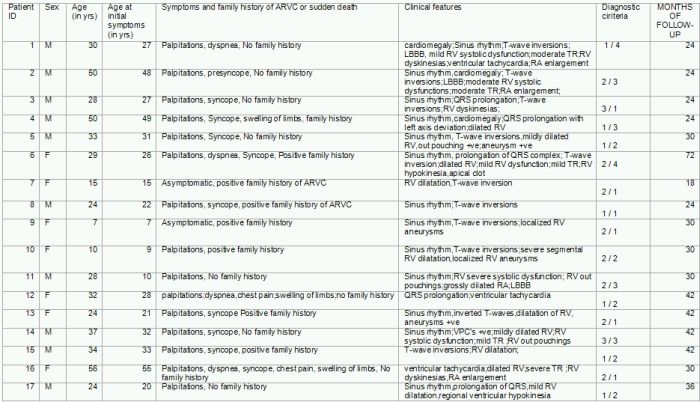

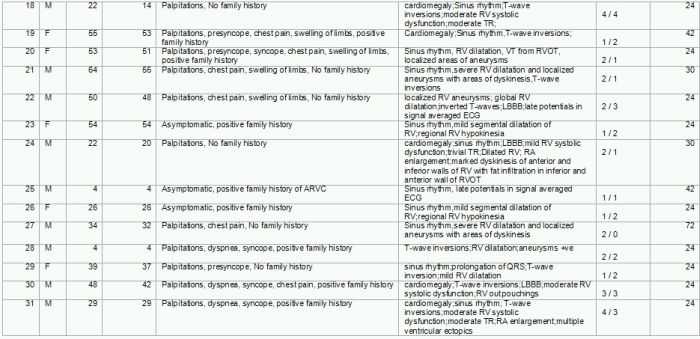
Symptoms and clinical features of all the patients included in the study

**Table 2:**
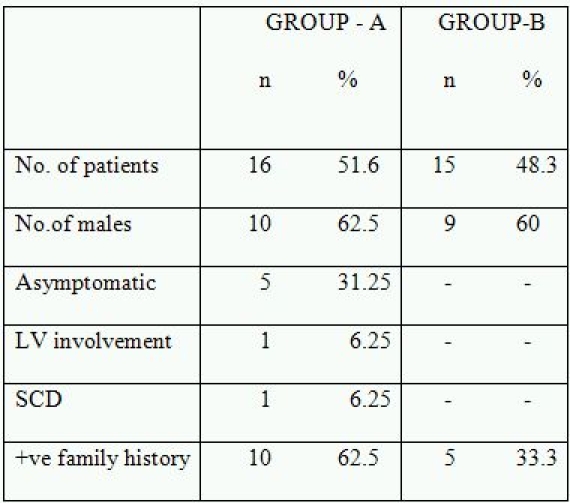
Gives the comparison of ARVD/C in Group A (≤30 years) and Group B (>30 years)

P=0.266683448
